# Evaluation of hemodynamic goal-directed therapy to reduce the incidence of bone cement implantation syndrome in patients undergoing cemented hip arthroplasty – a randomized parallel-arm trial

**DOI:** 10.1186/s12871-018-0526-4

**Published:** 2018-06-06

**Authors:** Kai B. Kaufmann, Wolfgang Baar, Judith Rexer, Thomas Loeffler, Sebastian Heinrich, Lukas Konstantinidis, Hartmut Buerkle, Ulrich Goebel

**Affiliations:** 1 Department of Anaesthesiology and Critical Care, Medical Center – University of Freiburg, Hugstetter Strasse 55, 79106 Freiburg, Germany; 2 Department of Orthopaedic and Trauma Surgery, Medical Center – University of Freiburg, Freiburg, Germany; 3 Faculty of Medicine, University of Freiburg, Freiburg, Germany

**Keywords:** Hip arthroplasty, Postoperative complications, Hemodynamics

## Abstract

**Background:**

The bone cement implantation syndrome (BCIS) is a frequent and potentially disastrous intraoperative complication in patients undergoing cemented hip arthroplasty. Several risk factors have been identified, however randomized controlled trials to reduce the incidence of BCIS are still pending. We hypothesized that goal-directed hemodynamic therapy guided by esophageal Doppler monitoring (EDM) may reduce the incidence of BCIS in a randomized, controlled parallel-arm trial.

**Methods:**

After approval of the local ethics committee, 90 patients scheduled for cemented hip arthroplasty at the Medical Center – University of Freiburg were randomly assigned to either standard hemodynamic management or goal-directed therapy (GDT) guided by an esophageal Doppler monitoring-based algorithm. The primary endpoint was the incidence of overall BCIS including grade 1–3 after cementation of the femoral stem. Secondary endpoints included cardiac function, length of hospital stay and postoperative complications.

**Results:**

Ninety patients were finally analyzed. With regards to the primary endpoint, the overall incidence of BCIS showed no difference between the GDT and control group. Compared to the control group, patients of the GDT group showed a higher cardiac index before and after bone cement implantation (2.7 vs. 2.2 [l●min^− 1^●m^− 2^]; 2.8 vs. 2.4 [l●min^− 1^●m^− 2^]; *P* = 0.003, *P* = 0.042), whereas intraoperative amount of fluids and mean arterial pressure did not differ.

**Conclusions:**

The implementation of a specific hemodynamic goal-directed therapy did not reduce the overall incidence of BCIS in patients undergoing cemented hip arthroplasty.

**Trial registration:**

This randomized clinical two-arm parallel study was approved by the local Ethics Committee, Freiburg, Germany [EK 160/15, PI: U. Goebel] and registered in the German Clinical Trials Register (DRKS No. 00008778, 16th of June, 2015).

## Background

The number of hemiarthroplasties to treat femoral neck fractures or total hip replacement surgery due to osteoarthritis is increasing steadily while patients’ age and severe comorbidities increase concomitantly [1, 2]. For several reasons orthopedic surgeons prefer cemented hip arthroplasty mainly for older patients reducing the rate of re-operation due to aseptic loosening [3]. Although safety guidelines for the reduction of bone cement implantation syndrome (BCIS) have been published recently [4], BCIS remains a frequent intraoperative complication with an overall incidence of up to 28% [5]. Up to date, the pathophysiology is not fully understood [5]. The main clinical problem is an acute right ventricular insufficiency due to pulmonary embolism caused by fat tissue and bone cement [6–8]. Donaldson and colleagues established a severity classification of BCIS according to oxygen desaturation and hypotension (Table 1) [6]. Patients with BCIS grade 2 and 3 showed a 16-fold increase in 30 day postoperative mortality compared to those with BCIS grade 1 [5, 9]. Several independent predictors for severe BCIS have been identified: ASA grade 3–4, chronic obstructive pulmonary disease and preoperative medication with diuretics or warfarin [5].

**Table 1:** Definition of Bone cement implantation syndrome (BCIS) [6] and primary endpoint

BCIS grade 1	Moderate hypoxia (SpO_2_ < 94%) or hypotension (fall in systolic blood pressure (SBP) > 20%)		
BCIS grade 2	Severe hypoxia (SpO_2_ < 88%) or hypotension (fall in SBP > 40%) or unexpected loss of consciousness		
BCIS grade 3	Cardiovascular collapse requiring CPR		
BCIS grade	GDT (*n* = 45)	Control (n = 45)	*P* Value
1	17 (38)	14 (31)	0.51
2	0 (0)	7 (16)	*0.006*
3	0	0	
Total	17 (38)	21 (47)	0.39

Data are presented as number of patients and percentage within the group. Results with a *P *Value < 0.05 are considered significant and are written in italic type

*GDT* goal-directed therapy

A variety of studies with promising results have been conducted on a goal-directed hemodynamic therapy for patients scheduled for total hip replacement or repair of femoral neck fractures [10–12]. According to these studies esophageal Doppler monitor-guided (EDM) goal-directed therapy (GDT) shortened time to being medically fit for discharge and resulted in a significantly reduced length of hospital stay [10, 12]. However, these studies did not focus on the incidence of BCIS. The Enhanced Recovery after Surgery (ERAS®) Society developed an evidence-based algorithm for goal-directed hemodynamic management which includes monitoring of cardiac output and optimization of stroke volume [13, 14]. Different hemodynamic monitoring methods (e.g., pulse contour method, pulmonary artery catheter, transesophageal echocardiography) have been used to guide individualized intraoperative management [15]. However, compared to the esophageal Doppler technique most of these methods are invasive, bearing an increased risk for complications [16–18]. Whereas the accuracy of EDM was validated in various studies [19]. The effect of EDM GDT on the incidence of BCIS during cemented hip arthroplasty has not been investigated.

The hypothesis of this study was that optimizing hemodynamic parameters by EDM guided GDT before cement implantation would reduce the incidence of BCIS.

## Methods

This randomized clinical two-arm parallel study was approved by the local Ethics Committee, Freiburg, Germany [EK 160/15, PI: U. Goebel] and registered in the German Clinical Trials Register (DRKS No. 00008778, 16th of June, 2015). The study was conducted at the Department of Anesthesiology and Intensive Care and the Department of Orthopedic Surgery, Medical Center – University of Freiburg, Faculty of Medicine, University of Freiburg, Germany.

**Fig. 1:**
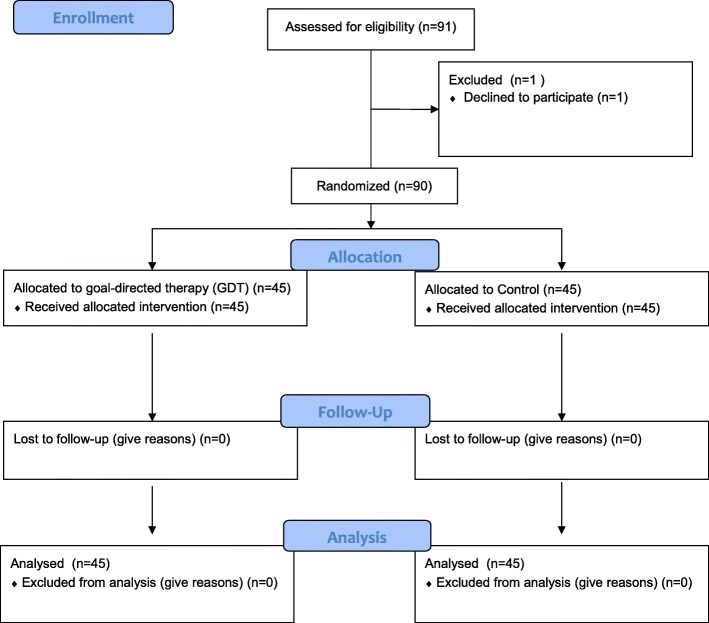
CONSORT Statement

Patients admitted to the Department of Orthopedic Surgery at the Medical Center University of Freiburg between September 2015 and October 2016 were eligible for this study (Fig. 1). Inclusion criteria were cemented hip arthroplasty under general anesthesia, age > 18 years and written informed consent. Exclusion criteria were body-mass-index (BMI) > 50, esophageal- or gastric pathologies and pregnancy.

### Anesthetic protocol

All patients enrolled in this study received a femoral nerve block in single shot technique before general anesthesia was started. This procedure was ultrasound-guided (in-plane approach) and conducted via a 22 G needle (Pajunk, Geisingen, Germany). In total, a single dose of 20 ml ropivacaine 0.2% was administered. Patients’ radial artery was cannulated for continuous blood pressure and intermittent blood gas analysis. In patients with ASA classification ≥3 a central venous line via the internal jugular vein was established. Anesthesia was induced with intravenous sufentanil 0.5μg•kg^− 1^ and target-controlled infusion of propofol at plasma concentrations of 2.5–4 μg•ml^− 1^ (Propofol 1% MCT and Injectomat TIVA Agilia, Fresenius-Kabi GmbH, Bad Homburg, Germany). If patients had a history of postoperative nausea and vomiting propofol was used to maintain general anesthesia. Otherwise desflurane was used at a minimal alveolar concentration (MAC) of 0.9. To facilitate endotracheal intubation an intravenous bolus of cis-atracurium (0.1 mg•kg BW^− 1^) was administered. During surgery, the grade of muscle relaxation was monitored using the quantitative and qualitative train-of-four technique. All patients were successfully extubated in the operating room and then transferred to the intensive care unit afterwards. The medical and nursing staff taking care of the patients on the ward were blinded to group assignment.

### Study design

**Fig. 2:**
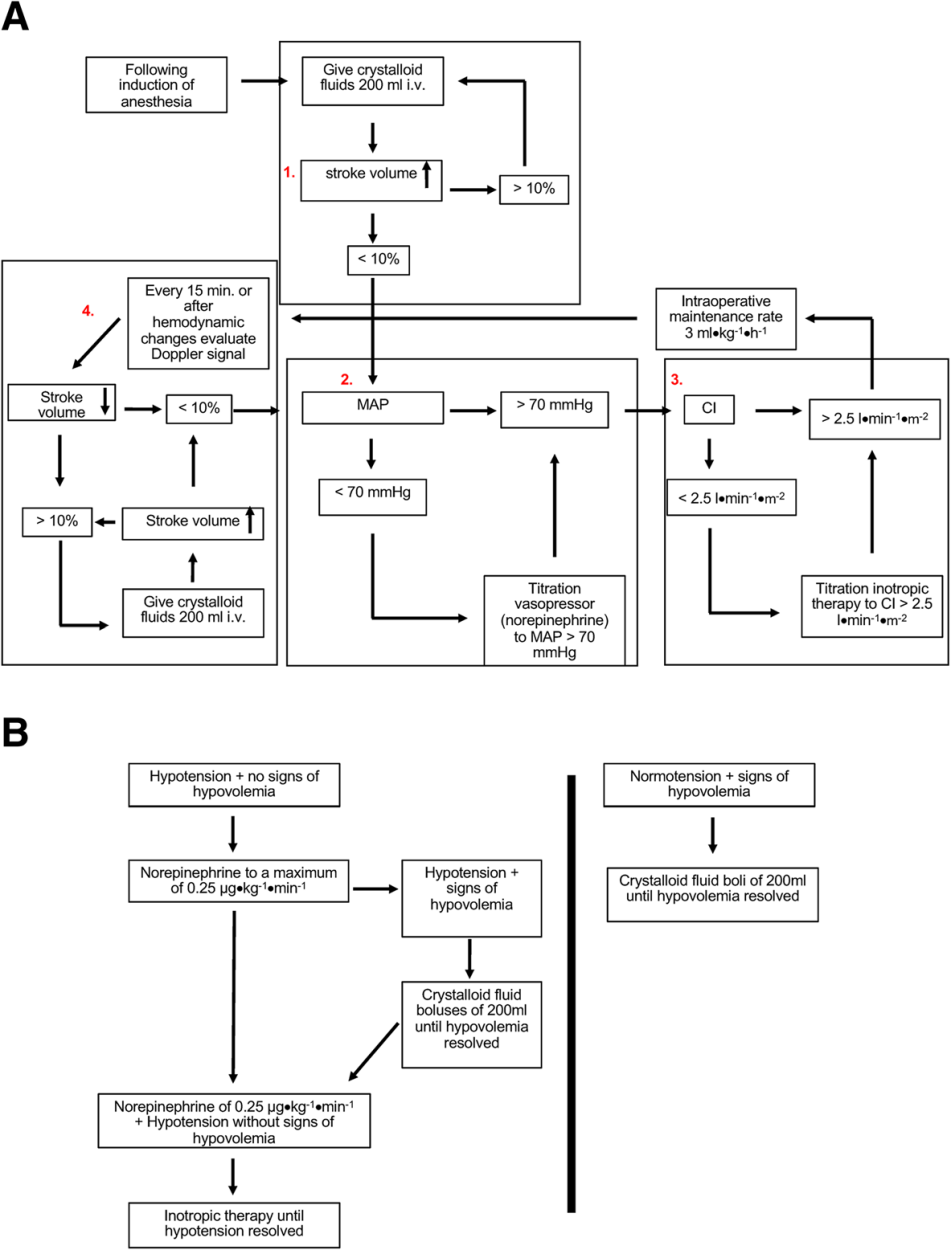
(**a**) Goal-directed hemodynamic therapy (GDT) (**b**) Standard hemodynamic algorithm. (**a**) Goal-directed hemodynamic algorithm to guide intraoperative volume, vasopressor and inotropic therapy in the Goal-directed therapy group adopted from ERAS [13]. (**b**) Hemodynamic algorithm to guide intraoperative volume, vasopressor and inotropic therapy in the control group according to the standard operating procedure of the department of orthopedic anesthesia

Based on a computer-generated list, patients were randomly allocated (using the sequentially numbered, opaque, sealed envelope technique) to one of the following two groups after induction of general anesthesia: (A) goal-directed therapy (GDT) group based on an EDM-guided algorithm (Fig. 2a); (B) control group with conventional fluid and hemodynamic management along standard operating procedures of the department of orthopedic anesthesia (Fig. 2b). The allocation ratio was 1:1. Written informed consent was obtained from all patients prior to enrolling them in the study. The nature of the study did not allow complete blinding of anesthesiologists. The anesthesiologist in charge treating the patient in the operating room had to stick either to the ERAS algorithm or to the control group management, so blinding in the operating room was not possible.

In both groups a Doppler probe was placed in the mid esophagus immediately after induction of anesthesia monitored by aortic visual and auditory signals (Deltex Medical™, Chichester, United Kingdom). In patients of the control group, EDM (displaying stroke volume and cardiac index) was covered and made invisible to the treating anesthesiologist. In both groups intraoperative maintenance rate of crystalloid fluids was set to 3 ml●kg^− 1^●h^− 1^. EDM-derived hemodynamic data were analyzed at four time periods: before start of surgery, before bone cement insertion, after bone cement implantation into the femur and at the end of surgery. All EDM variables, including cardiac index, stroke volume, stroke distance, flow time, mean acceleration, peak velocity, heart rate and systemic arterial pressure were recorded every 5 s.

Patients randomized to the GDT group were managed according to the ERAS® algorithm utilizing EDM variables (i.e., MAP, stroke volume and cardiac index) (Fig. 2a) Following induction of anesthesia a bolus of 200 ml of crystalloid fluid was administered. If stroke volume increased by ≥10%, additional boli of 200 ml were given until the increase in stroke volume was less than 10%. As long as MAP was less than 70 mmHg and/or cardiac index (CI) less than 2.5 l●min^− 1^●m^− 2^ despite an increase in stroke volume of less than 10% following fluid challenge, low dose norepinephrine by continuous infusion and/or ephedrine were administered, respectively. Fluid responsiveness and hemodynamic variables were re-assessed at least every 15 min and more frequently in case of hemodynamic instability or bone cement insertion into the femur (Fig. 2a).

Patients allocated to the control group were hemodynamically managed as follows. Isolated hypotension (defined as 20% decrease in mean arterial blood pressure (MAP) below baseline or less than 60 mmHg) was treated by a continuous infusion of norepinephrine up to a maximum rate of 0.25 μg●kg^− 1^●min^− 1^. If hypotension persisted, inotropes were administered until MAP was ≥60 mmHg. In the control group the type of inotropic therapy was not predefined according to a study protocol. Therefore, dobutamine, epinephrine, and ephedrine were used according to individual preferences. If hypotension was accompanied by signs of hypovolemia (defined as urine output less than 0.5 ml●kg^− 1^●h^− 1^ and/or an increase in heart rate of more than 20% above baseline) crystalloid fluid was administered until urine output and/or heart rate normalized. If hypotension persisted despite volume challenges, norepinephrine was administered at the above listed dosage (Fig. 2b).

### Outcome measures

The primary endpoint was the incidence of BCIS as defined by Donaldson and colleagues (Table 1) [6]. From the start of our study, including the a-priori power analysis for sample size calculation we focused on the incidence of BCIS applying a standardized severity classification to detect differences in hemodynamics after bone cement implantation between the GDT and control group. The aim was to have patients’ hemodynamic parameters optimized to the maximum at the time of cement implantation. This included the following key points which were optimized before the implantation of bone cement into the femur: preload, cardiac performance and mean arterial pressure. Our intention was to show that due to the applied hemodynamic algorithm, patients in the GDT group showed significantly improved hemodynamic parameters compared to the control group before bone cement insertion into the femur. We hypothesized that this significant, preemptive hemodynamic optimization due to the EDM guided algorithm reduces the risk of BCIS. Mean arterial pressure and cardiac performance was recorded every 5 s. As described by Olsen and colleagues only periods of hypotension and oxygen desaturation that occurred within 15 min after bone cement insertion into the femur were considered for the diagnosis of BCIS [5].

Secondary endpoints included acute kidney injury (as defined by the Acute Kidney Injury Network Criteria), cardiac morbidity (myocardial infarction as diagnosed by electrocardiogram and/or troponin T serum concentration; newly developed atrial fibrillation), neurological morbidity (stroke and delirium), wound healing disorder (drainage of pus from surgical wound) and pulmonary complications (respiratory infection, respiratory failure, pleural effusion, atelectasis, pneumothorax, bronchospasm, aspiration pneumonitis) [20]. Length of hospital stay was documented. Secondary endpoints were re-assessed within 30 days after surgery. All outcomes were documented by research personnel unaware of patients’ group assignment.

### Statistical analysis

The sample size calculation was based on a previously reported overall incidence of BCIS during cemented hip arthroplasty of approximately 28% [5]. Due to the clinical experience of our department of orthopedic anesthesia with a low incidence of overall BCIS of 5% for patients with cemented hip arthroplasty we calculated a sample size of 45 patients per group with a power of 80% and a two-sided significance level of 5%. We hypothesized that this low incidence of BCIS was the result of a specific hemodynamic goal-directed therapy guided by EDM. We used the incidence of overall BCIS for sample size calculation, aware that this study is under-powered to draw conclusions on results about BCIS grade 2 and 3 which are post hoc subgroup findings. Categorical data were analyzed using Fisher’s exact test. Continuous variables were examined for normal distribution. If data were normally distributed, independent samples t-test for normally distributed variables was performed. In case of not normally distributed data, the Mann-Whitney U test was used. Statistical analysis was performed using IBM™, (Armonk, United States) SPSS Statistics 22 software.

## Results

The consort diagram is shown in Fig. 1. Ninety-one patients were assessed for eligibility between September 2015 and October 2016. One patient was excluded because formal informed consent was withdrawn. Ninety patients were randomly allocated to the goal-directed (GDT) or control group. Finally, 45 patients were analyzed in each group. No adverse events occurred due to EDM probe placement.

### Primary endpoint

The overall incidence of BCIS did not differ significantly between the GDT and control group (Table 1). Post hoc subgroup analysis showed a difference for BCIS grade 2. In the GDT group, none of the patients showed BCIS grade 2 whereas 7 patients (16%) of the control group did (*P* = 0.006).

### Secondary endpoints

The incidences of postoperative wound healing disorder, neurological, pulmonary or cardiac complications were comparable between groups. One patient (2%) of the GDT group compared to 6 patients (13%) of the control group developed postoperative acute kidney injury (*P* = 0.049). With regards to the incidence of overall complications there was a trend of less complications in the GDT group (Table 2).

**Table 2:** Postoperative complications

	GDT (n = 45)	Control (*n* = 45)	*P* Value
Acute kidney injury	1 (2)	6 (13)	*0.049*
Neurological	3 (7)	2 (4)	0.65
Wound healing disorder	1 (2)	3 (7)	0.31
Pulmonary	7 (16)	7 (16)	1.0
Cardiac	0	1 (2)	0.32
Total	10 (22)	17 (38)	0.11

Data are presented as number of patients and percentage within the group

*GDT* goal-directed therapy. Results with a *P* Value < 0.05 are considered significant and are written in italic type

### Baseline patient characteristics and perioperative parameters

Despite all other parameters, the number of patients with chronic kidney disease stage 3 diagnosed by a preoperative glomerular filtration ratio of 30 to 60 ml differed significantly between both groups (Table 3).

**Table 3:** Baseline patient characteristics

	GDT (n = 45)	Control (*n* = 45)	*P* Value
Male/female	33% / 66%	44% / 56%	0.28
Age	79 (74.5–83.5)	79 (72.5–83)	0.61
Body mass index	24.8 (22.3–28.3)	24.6 (21.4–26.8)	0.44
ASA 1	0%	0%	
ASA 2	17 (38)	10 (22)	0.11
ASA 3	23 (51)	28 (62)	0.29
ASA 4	5 (11)	7 (16)	0.54
CAD	5 (11)	10 (22)	0.16
Hypertension	30 (67)	24 (53)	0.2
Liver disease	0	1 (2)	0.32
GFR > 90 ml	4 (9)	7 (16)	0.33
GFR 60 - 90 ml	11 (24)	14 (31)	0.48
GFR 30 - 60 ml	28 (62)	17 (38)	*0.02*
GFR 15 - 30 ml	2 (4)	7 (16)	0.08
Diabetes	9 (20)	9 (20)	1.00
Beta receptor blocker	14 (31)	19 (42)	0.27
Anticoagulation	18 (40)	18 (40)	1.00
Acetylsalicyl acid	9 (20)	12 (27)	0.46
ACE inhibitor	13 (29)	11 (24)	0.63
ARBs	8 (18)	12 (27)	0.31
Diuretics	11 (24)	15 (33)	0.35
Nicotine dependence	3 (7)	5 (11)	0.46
Indication for surgery:			
Trauma	20 (44)	19 (42)	0.83
Arthrosis	8 (36)	8 (36)	1.00
Infectious	6 (13)	7 (16)	0.76
Necrosis	3 (7)	0 (0)	0.08
Osteolysis	0 (0)	3 (7)	0.08

Data are presented as number of patients and percentage within the group or median and interquartile range

*ASA* American Society of Anaesthesiologists, *GDT* goal-directed therapy, *CAD* coronary artery disease, *ACE inhibitor* angiotensin-converting enzyme inhibitor, *ARBs* Angiotensin-receptor-II blockers, *GFR* glomerular filtration ratio.  Results with a *P* Value < 0.05 are considered significant and are written in italic type

Anesthesia and surgery time intervals, total blood loss, urinary excretion and administered crystalloids were comparable between the groups. With regards to the intraoperative catecholamine therapy the two groups showed significant differences. Patients of the GDT group had less norepinephrine but received inotropic medication more often (Table 4). The need for vasopressor therapy after surgery was comparable between both groups. In addition, length of hospital stay was also comparable between groups (Table 4).

**Table 4:** Perioperative data

	GDT (n = 45)	Control (n = 45)	*P* Value
Anesthesia (min)	208 ± 57	231 ± 66	0.09
Surgery (min)	117 ± 42	127 ± 55	0.34
Blood loss (ml)	504 ± 407	410 ± 354	0.29
Urinary excretion (ml●kg^−1^●h^−1^)	2.9 ± 2.7	3.3 ± 2.2	0.60
Crystalloids (ml●kg^− 1^●h^− 1^)	13 ± 5	13 ± 5	0.42
Norepinephrine (μg●kg^− 1^●min^− 1^)	0.03 ± 0.03	0.05 ± 0.03	0.05
Norepinephrine max (μg●kg^− 1^●min^− 1^)	0.05 ± 0.03	0.08 ± 0.04	*0.003*
Inotropics	34 (76)	23 (52)	*0.02*
Vasopressors within 24 h postop	5 (11)	11 (26)	0.08
Vasopressors within 72 h postop	2 (4)	5 (12)	0.21
Length of hospital stay (days)	17 ± 7	19 ± 9	0.22

Data are presented as number of patients and percentage or mean and standard deviation

*GDT* goal-directed therapy. Results with a *P* Value < 0.05 are considered significant and are written in italic type

### Intraoperative hemodynamic parameters

For all four time periods mean acceleration (MA), heart rate (HR), peak velocity (PV), systolic (BP sys) and mean arterial pressure (MAP) did not differ between groups. Patients of the GDT group showed an increased stroke distance and flow time prior to cement implantation. The cardiac index and stroke volume index before, after and at the end of surgery differed between the GDT and control group although this difference was not present at the beginning of surgery (Table 5).

**Table 5:** Intraoperative hemodynamic parameters

		GDT (n = 45)	Control (n = 45)	*P* Value
SD (cm)	pre	9.9 ± 3.11	9.5 ± 2.9	0.49
	before	10.4 ± 2.9	9.0 ± 2.9	*0.02*
	after	10.1 ± 2.9	8.9 ± 3.1	0.07
	end	9.6 ± 3.1	8.6 ± 3.1	0.12
FTc (ms)	pre	293 ± 42	290 ± 44	0.69
	before	303 ± 37	285 ± 41	*0.03*
	after	298 ± 44	279 ± 40	*0.04*
	end	289 ± 49	271 ± 44	0.07
MA (cm●s^−2^)	pre	6.4 ± 2.5	6.3 ± 2.0	0.77
	before	6.3 ± 2.0	6.4 ± 2.0	0.78
	after	6.7 ± 2.0	6.9 ± 2.5	0.61
	end	7.1 ± 2.2	7.5 ± 2.5	0.52
CI (l●min^−1^●m^− 2^)	pre	2.6 ± 0.8	2.4 ± 0.9	0.30
	before	2.7 ± 0.7	2.2 ± 0.7	*0.003*
	after	2.8 ± 0.8	2.4 ± 1.1	*0.04*
	end	2.7 ± 0.9	2.3 ± 0.9	*0.04*
SVI (ml●m^−2^)	pre	44 ± 1	42 ± 15	0.35
	before	47 ± 13	40 ± 15	*0.02*
	after	45 ± 12	39 ± 16	*0.04*
	end	43 ± 13	37 ± 16	0.07
HR (1●min^−1^)	pre	59 ± 14	58 ± 10	0.60
	before	61 ± 13	61 ± 9	0.99
	after	64 ± 14	63 ± 11	0.54
	end	66 ± 15	64 ± 11	0.39
PV (cm●s^−1^)	pre	50 ± 14	49 ± 14	0.76
	before	50 ± 12	48 ± 15	0.43
	after	51 ± 12	49 ± 16	0.49
	end	51 ± 14	50 ± 16	0.64
BP sys (mmHg)	pre	124 ± 16	119 ± 14	0.12
	before	129 ± 15	126 ± 13	0.24
	after	134 ± 16	129 ± 18	0.15
	end	122 ± 14	120 ± 15	0.43
MAP (mmHg)	pre	82 ± 12	81 ± 11	0.70
	before	85 ± 11	85 ± 10	0.90
	after	87 ± 12	86 ± 11	0.75
	end	80 ± 9	80 ± 10	0.84

Data are presented as mean and standard deviation

*GDT* Goal-directed Therapy, *MAP* mean arterial pressure, *HR* heart rate, *SD* stroke distance, *FTc* corrected flow time, *CI* cardiac index, *SVI* stroke volume index, *PV* peak velocity, *BP sys* systolic blood pressure, *BP dia* diastolic blood pressure, *pre* before surgical incision, *before* before bone cement insertion, *after* after bone cement insertion, *end* end of surgery. Results with a *P* Value < 0.05 are considered significant and are written in italic type

## Discussion

The main results of this study can be summarized as follows: First, EDM GDT did not reduce the rate of overall BCIS including grade 1–3. Post hoc subgroup analysis showed a reduction of BCIS grade 2 in the GDT group. Second, due to EDM GDT, patients showed better cardiac performance with no differences in MAP by using more inotropes, less vasopressors but the same amount of fluids. Third, despite an increased number of patients with preoperative renal dysfunction in the GDT group according to the baseline characteristics, these patients showed a decreased incidence of postoperative acute kidney injury.

The overall incidence of BCIS in this study is higher than described in current literature [5]. The reason for this discrepancy could be the overall increased systolic blood pressure just before cement implantation which was obvious in both groups as a result of volume or catecholamine therapy. Communication with the surgical team at the moment of bone cement implantation is essential in the management of BCIS and is part of our standard operating procedure independent of study group assignment. This may explain the mentioned iatrogenic increase in systolic blood pressure immediately before bone cement implantation in this study. Thus, a decrease of systolic blood pressure to diagnose BCIS might become more obvious. Another reason for this discrepancy might be that Olsen and colleagues collected data from a chart review on systolic blood pressure every fifth minute for a period of at least 15 min after bone cement implantation. In this randomized trial, systolic blood pressure was recorded every five seconds continuously so that our approach is more sensitive.

Although BCIS is described as a multifactorial phenomenon the acute right ventricular dysfunction due to pulmonary embolism is a key feature [6, 21]. Compared to the control group EDM in the GDT group provides insights into cardiac function; i.e. hemodynamic parameters. The aim was to have patients’ hemodynamic parameters optimized to the maximum at the time of cement implantation. This included the following key points which were optimized before bone cement implantation: preload, cardiac performance and mean arterial pressure. We hypothesized that EDM GDT leads to a pre-emptive optimization of patients’ hemodynamic status, reducing the risk for severe BCIS. Patients of the GDT group experienced an individually tailored preloading with crystalloid fluids according to stroke volume optimization without risking volume overload. Low cardiac output syndrome was avoided by additional use of inotropic therapy also supporting the right ventricle. Apart from that, acute increase of pulmonary vascular resistance as part of BCIS might lead to distension of the right ventricle with a concomitant decrease of coronary blood flow. Therefore keeping up a sufficient systemic blood pressure is crucial [6–8]. This aim was achieved by norepinephrine administration. The results show that although not different at the beginning, EDM GDT led to better cardiac and stroke volume index at the time of cement insertion while mean arterial pressure was comparable between groups. Although cardiac and stroke volume index were significantly higher in the GDT group we were not able to reduce the risk for overall BCIS.

Most studies conducted on intraoperative goal-directed fluid management focused on abdominal surgery using colloids to optimize stroke volume. Inconsistent results were reported [22–27]. In contrast to these studies we did not use colloids to optimize stroke volume due to several known side effects caused by liberal administration. In accordance with current literature we used a combination of goal-directed fluid therapy and inotropic medication instead of single goal-directed fluid therapy [13, 14, 28–30]. Data on EDM-guided goal-directed fluid therapy in orthopedic surgery showed promising results with regards to length of hospital stay [10, 12]. However, those studies did not focus on patients undergoing cemented hip arthroplasty. In this randomized controlled trial the amount of intraoperative crystalloid fluids were comparable between groups, whereas the GDT group received inotropic medication more frequent but less norepinephrine. Timing of stroke volume optimization with crystalloids seems to be crucial as only 20% is kept intravascular [31]. The timing of fluid boluses due to different triggers was different between the groups. As a consequence, the overall amount of fluids showed no difference between the groups whereas a different timing led to better cardiac performance in the GDT group. With regards to the difference in catecholamine therapy there was no difference in mean arterial pressure at any time between the groups, although hemodynamic variables differed significantly. This result underlined the fact that norepinephrine primarily acts as a vasopressor leading to an increased afterload, that might in part be responsible for a decreased cardiac performance (cardiac and stroke volume index).

With regards to already known independent predictors of BCIS (ASA ≥ 3, femoral neck fracture, medication with diuretics and anticoagulation) randomization in this study appears adequate [5, 9, 32]. The high percentage of patients classified as ASA 3 and 4 in this study makes the data presented reliable. The only baseline parameter that differed between groups was the number of patients suffering from chronic kidney disease grade 3 which was significantly higher in the GDT group. Nevertheless, regarding the secondary endpoints the incidence of postoperative acute kidney injury was significantly higher in the control group.

This study has several limitations. A limitation referring to the esophageal Doppler technique to measure stroke volume and cardiac index is the assumption and not individualized measurement of the aortic diameter derived from patients’ height and weight. As a result, over- and underestimation of stroke volume can occur due to anatomical anomalies of the descending aorta [19]. Like most of the hemodynamic monitoring devices apart from the pulmonary artery catheter (which is an invasive technique with an increased risk for side effects), EDM is not suitable to distinguish between right or left ventricular dysfunction [17]. In agreement with findings of previous studies, postoperative complications, especially the incidence of acute kidney injury was reduced in the GDT compared to the control group [10, 12, 22]. These findings on renal morbidities must be viewed with caution because the study was not sufficiently powered to allow reliable statistical assessment of these outcome variables. Our aim was to detect a reduction of overall BCIS from 28 to 5%, which appears quite optimistic, but due to our a priori clinical experience using this EDM GDT for cemented hip arthroplasties this approach did not appear unrealistic. As a consequence, this study was only powered to detect a difference in the incidence of overall BCIS including grade 1–3 between the GDT and control group. This study is under-powered to draw reliable conclusions regarding the incidence of BCIS grade 2.

## Conclusions

In summary, this is the first randomized controlled trial that focuses on the prevention of BCIS in multimorbid patients who are at increased risk using a semi-invasive technique for a differentiated hemodynamic management. The implementation of EDM GDT in patients undergoing cemented hip arthroplasty was not the reason for the low incidence of BCIS at our department. We were not able to reduce the overall incidence of BCIS by this specific EDM GDT. This trial should be considered as a pilot study for further clinical trials including more medical centers to validate a possible risk reduction for BCIS including more intraoperative variables.

## Abbreviations

ACE inhibitor: angiotensin-converting enzyme inhibitor
ARBs: Angiotensin-receptor-II blockers
ASA: American Society of Anesthesiologists
BCIS: Bone cement implantation syndrome
BP dia: diastolic blood pressure
BP sys: systolic blood pressure
CAD: coronary artery disease
CI: Cardiac index
EDM: Esophageal Doppler monitoring
FTc: corrected flow time
GDT: goal-directed therapy
GFR: glomerular filtration ratio
HR: heart rate
MAP: Mean arterial pressure
PV: peak velocity
SD: stroke distance
SVI: stroke volume index
